# Correlation between FNDC5 expression and tau pathology in elderly individuals

**DOI:** 10.1002/alz.087798

**Published:** 2025-01-03

**Authors:** Ícaro Raony, Mychael V Lourenco

**Affiliations:** ^1^ Antonio Pedro University Hospital, Fluminense Federal University, Niterói, Rio de Janeiro Brazil; ^2^ Institute of Medical Biochemistry Leopoldo de Meis, Federal University of Rio de Janeiro, Rio de Janeiro, Rio de Janeiro Brazil; ^3^ Institute of Medical Biochemistry Leopoldo de Meis, Federal University of Rio de Janeiro, Rio De Janeiro, Rio de Janeiro Brazil

## Abstract

**Background:**

Physical exercise has been proposed as an approach to reduce the risk of Alzheimer’s disease (AD). Engaging in physical exercise triggers the shedding of the extracellular domain of fibronectin type III domain‐containing protein 5 (FNDC5), producing a circulating peptide (irisin) that promotes neuroprotection in AD mouse models. Despite recent evidence indicating that reduced FNDC5/irisin levels in brain and cerebrospinal fluid correlate with amyloid beta pathology, the impact of FNDC5/irisin on tau pathology remains uncertain. Here, we investigated the correlation between brain FNDC5 expression and key indicators of tau pathology in a cohort of elderly subjects.

**Method:**

FNDC5 expression and tau pathology were examined in the postmortem hippocampus of elderly subjects enrolled in The Aging, Dementia and Traumatic Brain Injury (TBI) Study (https://aging.brain‐map.org). FNDC5 expression data were obtained from RNA sequencing, whereas tau pathology was evaluated using the Braak neuropathological scale. Levels of pThr181‐tau and total tau were measured through Luminex assays, and AT8 (pSer202/Thr205 tau) was assessed by immunohistochemistry. Inclusion criteria included subjects (both males and females) aged over 77 years old, whereas exclusion criteria were presence of non‐AD dementia or a history of TBI diagnosis.

**Result:**

We observed that individuals aged over 90 years old exhibit a trend for lower hippocampal expression of FNDC5 compared to those ranging from 77‐89 years old (t = 1.87; p = 0.06). There was also a trend among individuals with high tau pathology (Braak III‐VI) showing reduced FNDC5 expression compared to those with low tau pathology (Braak I‐II) (t = 1.93, p = 0.06). Accordingly, reduced FNDC5 expression was associated with higher AT8‐positive labeling (Spearman r = −0.40; p = 0.01) and pThr181‐tau levels (Pearson r = −0.30; p = 0.07), with no correlations observed with total tau levels (Pearson r = 0.27; p = 0.11).

**Conclusion:**

These results suggest that FNDC5 expression in hippocampus is reduced with tau pathology in humans, encouraging further studies to explore how physical exercise and FNDC5/irisin may influence tau pathology during aging.

